# Current progress of labeling strategies in tissue clearing for large-scale biological visualization

**DOI:** 10.3389/fcell.2026.1886363

**Published:** 2026-08-13

**Authors:** Si-Wei Wang, Yu-Qian Wang, Jia-Wen Deng, Sheng-Bo Yang, Fang-Yuan Wang, Jun Wu, Jian-Guo Liu

**Affiliations:** 1 Key Laboratory of Oral Disease Research (Guizhou Provincial Department of Education & Zunyi City), School of Stomatology, Zunyi Medical University, Zunyi, China; 2 Department of Burn and Plastic Surgery, Medical Innovation Technology Transformation Center, Shenzhen Second People’s Hospital, The First Affiliated Hospital of Shenzhen University, Shenzhen, China; 3 Department of Human Anatomy, Zunyi Medical University, Zunyi, China

**Keywords:** 3D imaging, label-free imaging, labeling strategy, molecular labeling, optical clearing, tissue clearing

## Abstract

Tissue clearing (TC) has transformed three-dimensional (3D) imaging by enabling deep, volumetric visualization of intact organs and complex biological systems, yet its full potential is constrained by persistent challenges in molecular labeling, including limited penetration, heterogeneous signal distribution, and compromised specificity under harsh clearing conditions. In this review, we provide a systematic overview for labeling strategies in TC-based imaging, organized according to signal source into exogenous labeling, genetically encoded labeling, and label-free imaging approaches. We further delineate exogenous approaches into morphological, molecular, and functional labeling, reflecting a shift from anatomical mapping toward molecular and activity-resolved interrogation. Recent advances, including nanobody-based immunolabeling, bioorthogonal chemistry, activity-based probes, self-labeling enzyme tags, and intrinsic contrast imaging, are redefining the scope and resolution of cleared issue imaging. Beyond labeling modalities, we highlight cross-cutting innovation that enhances labeling performance across workflows, spanning chemical optimization, physical transport-assisted strategies, and emerging delivery platforms such as viral vectors and nanocarriers. Despite these advances, challenges persist in achieving rapid, homogeneous, and multiplexed labeling across large, heterogeneous, or clinically relevant specimens. By outlining these considerations, we aim to provide researchers with a clearer basis for selecting and optimizing labeling approaches within different TC workflows.

## 1 Introduction

Revealing the three-dimensional (3D) architecture of biological tissues from the cellular to the organ level provides a comprehensive understanding of the processes underlying organism physiology and pathophysiology (Richardson et al., 2021; Ertürk, 2024). Conventional histology, relying on labour-intensive sectioning, disrupts the native spatially intact tissue architecture and limits analysis to selected regions, potentially introducing bias while leaving much of the sample unexplored (Almagro et al., 2021). While clinical imaging methods, such as X-rays, computed tomography (CT) scans, and magnetic resonance imaging (MRI), facilitate the observation of entire organs, nevertheless, they are constrained in resolution for molecular and cellular/subcellular investigation (Liu et al., 2024; Deng et al., 2025). With advances in technology, tissue clearing (TC), which integrates labeling, volumetric imaging, and high-resolution data processing, emerged as one of the most revolutionary techniques of this century, bridging the gap between microscopic molecular visualization and macroscopic organ-level architecture from a mesoscale perspective (Ueda et al., 2020; Richardson et al., 2021; Ertürk, 2024).

**FIGURE 1:**
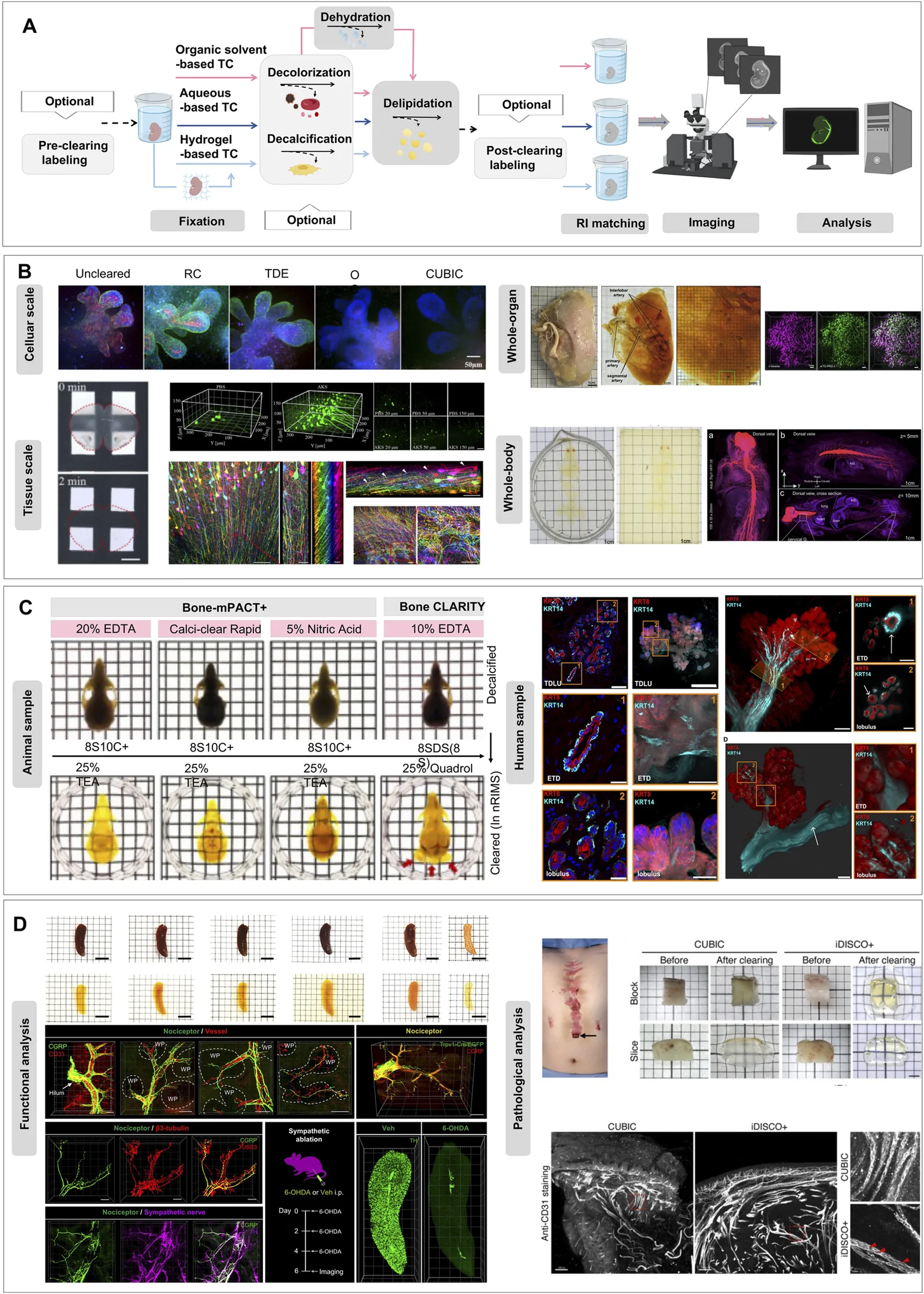
Tissue clearing (TC) workflows and applications. **(A)** Schematic overview of TC workflows. Three major TC methods are solvent-based, aqueous-based, and hydrogel-based TC, which share core modules including fixation, delipidation, RI matching, imaging and analysis. Optional steps involve decolorization, decalcification, dehydration, hydrogel embedded are incorporated in a method- or tissue-dependent manner. Labeling strategies may be applied either before or after clearing, depending on the properties of the probes and the specific requirements of the chosen TC protocol. **(B)** TC enables multiscales 3D imaging, spanning the cellular scale (e.g., organoids (Lallemant et al., 2020), reproduced under the terms of the CC-BY-NC license, Copyright 2020 POL Scientific), tissue scale (e.g., brain sections (Choquet et al., 2021) Copyright 2022 spring Nature), whole-organ scale (e.g., kidney (Zhao S. et al., 2020), reproduced under the terms of the CC BY-NC license, Copyright © 2020 Elsevier) to whole-body scale (e.g., mouse (Yi et al., 2024) Copyright 2024 spring Nature). **(C)** TC supports cross-species applications in both mouse and human samples (e.g., mouse skull (Jin et al., 2023), reproduced under the terms of the CC-BY-NC license, Copyright 2023 spring Nature, human breast (Paavolainen et al., 2024) Copyright 2024 Elsevier). **(D)** TC enables functional and pathological analyses, including detailed visualization of splenic neural immune architecture (Li et al., 2025) (Copyright 2025 American Physiological Society), and keloid vasculature (Nguyen et al., 2025) (Copyright 2025 Wiley). Illustration created with BioRender. Bone-mPACT+: Bone-modified passive tissue clearing techniques; CUBIC: clear, unobstructed brain imaging cocktails and computational analysis; EDTA: ethylenediaminetetraacetic acid; iDISCO: immunolabeling-enabled 3D imaging of solvent-cleared organs; TEA: triethanolamine; RI: refractive index; RC: RapiClear; TDE: 2,2′-thiodiethanol; OC: OptiClear; SDS:Sodium dodecyl sulfate; 8S10C (8% SDS and 10% SDC in 0.1 M PBS, pH 8.0).

The fundamental principle of TC is eliminating substantial molecules and pigments that cause light scattering and absorption within the tissue, along with refractive index (RI) matching, which facilitates deeper light penetration into the tissue (Richardson and Lichtman, 2015; Meng et al., 2025). Modern TC is classified into three types according to the chemical properties of the clearing reagents (Jing et al., 2019; Ueda et al., 2020; Meng et al., 2025) (Figure 1A): a) Organic solvent-based TC techniques, such as BABB (benzyl alcohol-benzyl benzoate) (Dodt et al., 2007), 3DISCO (three-dimensional imaging of solvent-cleared organs) and the developed DISCO series (Ertürk et al., 2012; Zhan et al., 2021 **)**, PEGASOS (polyethylene glycol (PEG)-associated solvent system) (Jing et al., 2018), enable rapid and efficient TC, providing superior transparency while accommodating tissue shrinkage; b) Aqueous reagent-based TC techniques, use hydrophilic chemicals such as urea, glycerol, and aminoalcohols to balance RI, providing enhanced fluorescence stability during clearing, although the process generally requires a relatively long incubation time. The representative methods as SeeDB (Ke et al., 2013) and CUBIC (clear, unobstructed brain imaging cocktails and computational analysis) (Susaki et al., 2014); c) Hydrogel-based TC techniques, including CLARITY (Chung et al., 2013), PACT (passive CLARITY technique) (Treweek et al., 2015), followed by Ce3D (Clearing-enhanced 3D), the diversity of these technologies embedding the tissue in the hydrogel facilitated effective and adaptable cleaning tailored to certain sample types. In general, these methods consist of several compatible modules, including sample fixation, optional pre-treatment (e.g., decolourization, decalcification, dehydration or hydrogel embedding), delipidation, fluorescent labeling, RI matching, image acquisition and analysis (Ueda et al., 2020; Richardson et al., 2021). Although ongoing optical TC techniques has enabled imaging of intact specimens and expanded their applications across a wide range of biomedical research, from the cellular level, tissue level to whole organs and even entire organisms (Dekkers et al., 2019; Lallemant et al., 2020; Puelles et al., 2021; Shan, et al., 2022; Sun et al., 2023; Yi et al., 2024; Yoshida et al., 2026), from rodent models to human biopsies (Blain et al., 2023; Jin et al., 2023; Eisenberg et al., 2024; Paavolainen et al., 2024; Thai et al., 2024; Horenberg et al., 2025), and from spatial anatomical reconstruction to *in situ* pathological analysis (Almagro et al., 2021; Chen et al., 2024; Paavolainen et al., 2024; Li et al., 2025; Nguyen et al., 2025), the labeling of specific cells and molecules throughout whole specimens remains a major bottleneck in TC applications (Tainaka et al., 2016; Ertürk, 2024) (Figures 1B–D).

To accurately phenotype individual cells and extract biologically meaningful information after TC, it is essential to use appropriate cell-labeling tools to visualize target molecules (Zhao et al., 2020; Richardson et al., 2021). Moreover, the performance of labeled target molecules abundance and efficiency critically influences the accuracy of quantitative analyses within the clearing framework (Streit et al., 2025). Despite the rapid advancements in TC and volumetric imaging, achieving deep, homogeneous, and target-specific labeling in large, optically cleared tissues is particularly challenging. Conventional labeling methods often suffer from limitations, including limited molecular diffusion, poor tissue penetration, prolonged incubation times, and fluorescence signal attenuation (Seo et al., 2016; Richardson et al., 2021).

Accordingly, there is a need for a comprehensive evaluation of labeling strategies and their optimization in the context of TC. Distinct from conventional classifications of TC methods, primarily based on chemical compositions or clearing principles, this review adopts a labeling-centred decision making driven by biological research needs. We systematically summarize current labeling strategies in TC, covering exogenous, genetically encoded, and label-free labeling, along with the chemical, physical, and delivery-based strategies developed to improve labeling efficiency. We also discuss the existing research gaps and potential directions for future development, aiming to provide a practical framework for selecting and implementing labeling strategies in 3D tissue imaging studies.

## 2 Labeling strategies by signal source

### 2.1 Exogenous labeling

**FIGURE 2:**
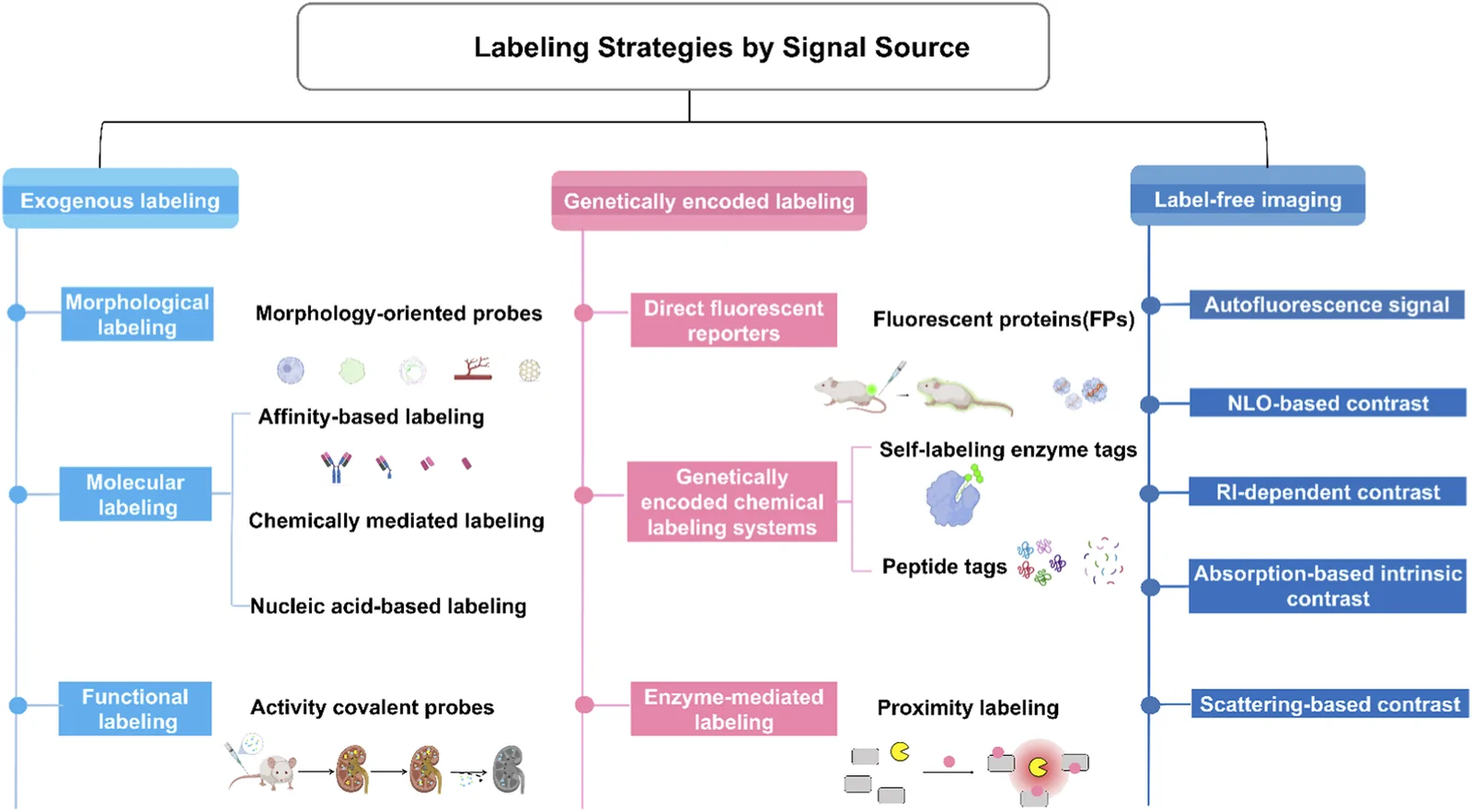
Labeling strategies by signal source of tissue clearing (TC)-based three-dimensional imaging. Current approaches can be broadly classified into exogenous labeling, genetically encoded labeling, and label-free imaging. Exogenous labeling includes morphological, molecular, and functional labeling using externally delivered probes. Genetically encoded labeling comprises direct fluorescent reporters, genetically encoded chemical labeling systems, and enzyme-mediated labeling approaches. Label-free imaging relies on intrinsic optical properties of tissues, including autofluorescence, nonlinear optical (NLO), refractive index (RI)-dependent, absorption-based, and scattering-based contrast mechanisms. Illustration created with BioRender.

In TC-based 3D imaging, exogenous labeling strategies use externally delivered probes to label endogenous biomolecules, typically without genetic encoding. Based on their biological information output, these strategies can be broadly classified into morphological labeling, molecular labeling, and functional labeling, reflecting a progression from anatomical visualization to molecular specificity and functional interrogation in 3D tissue imaging (Figure 2).

#### 2.1.1 Morphological labeling

Morphological labeling primarily aims to visualize cellular and tissue architecture and provide spatial context for volumetric reconstruction in cleared tissues. A variety of morphology-oriented probes provides versatile and rapid labeling of cellular and subcellular architectures (Dean and Palmer, 2014; Tainaka et al., 2016). Commonly used probes include nuclear dyes (DAPI, PI, TO-PRO3) (Mai et al., 2022; Harrison et al., 2025), lipophilic membrane dyes such as carbocyanine dyes (DiI, DiO, DiD) (Fumoto et al., 2020), protein- or cytoplasm-binding dyes (NHS esters, Eosin Y) (Laurino et al., 2023; Mao et al., 2020), bone mineral probes (Calcein, OsteoSense) (Pulous et al., 2022; Yi et al., 2019), among others. While diffusion-driven labeling enables rapid and homogeneous penetration across large tissue volumes, its effectiveness is highly dependent on compatibility with the harsh physicochemical conditions imposed by different TC protocols. Notably, the clearing process can induce conformational alterations in target biomolecules, potentially resulting in diminished probe binding or increased nonspecific interactions (Lai, et al., 2017).

#### 2.1.2 Molecular labeling

Molecular labeling aims to identify and localize specific biomolecules within cleared tissues, typically achieved through externally delivered probes that rely on non-covalent recognition or chemically mediated interactions.

##### 2.1.2.1 Affinity-based labeling

Immunolabeling has been a core method for visualizing the molecular architecture of tissues. Originating in the mid-20th century, the method was built on a principle: a primary antibody binds its endogenous antigen, and a secondary antibody selectively recognizes the host species or subclass of that primary antibody. This two-step arrangement not only amplifies signal but also permits multiplexed detection, enabling researchers to view several native proteins simultaneously in fixed tissues, or even in living systems when epitopes are exposed at the cell surface (Exbrayat, 2019; Choquet et al., 2021).

As TC technologies emerged, researchers quickly recognized the transformative potential of combining homogeneous immunolabeling with 3D volumetric imaging. Protocols such as iDISCO (immunolabeling-enabled 3D imaging of solvent-cleared organs) (Renier et al., 2014), SHANEL (Small-micelle-mediated human organ efficient clearing and labeling) (Mai et al., 2022), CLARITY (Chung et al., 2013), SUMIC (simple ultrafast multicolour immunolabelling and clearing) (Biswas et al., 2021), EMOVI (efficient tissue clearing and multi-organ volumetric imaging) (Hofmann, et al., 2021), expanded antibody staining from thin sections to entire organs and, in some cases, entire organisms. Yet this expansion also exposed the fundamental limitations of classical antibodies. With a molecular mass of ∼150 kDa, full-length antibodies diffuse slowly, struggle to cross heterogeneous tissue barriers, and often undergo degradation or precipitation during extended perfusion and clearing procedures. Even with optimized detergents and solvents, uniform penetration across multi-millimeter or centimeter-scale tissues remains a persistent challenge (Ertürk, 2024; Mino et al., 2024; Yamauchi et al., 2024).

Over the past few years, a series of technical breakthroughs has shifted this landscape, with an even more profound impact arising from the advent of nanobodies. The vDISCO (nanobody (VHH)-boosted 3D imaging of solvent-cleared organs) leveraged the 13-kDa VHH domain, nature’s smallest functional antigen-binding unit, to markedly accelerate tissue penetration and boost fluorescent protein signals in cleared specimens (Cai et al., 2023). By overcoming the diffusion bottleneck inherent to antibodies, vDISCO enabled a range of quantitative whole-body studies, from neuronal circuits mapping to immune-neural interactions (Cai et al., 2019), metastases (Pan et al., 2019), and mechanisms underlying cerebellar dysfunction (Özen et al., 2022). Moreover, a LYVE1-targeting nanobody was developed that demonstrated superior penetration and improved visualization of lymphatic vessels compared with conventional antibodies (Funk et al., 2025). A complementary POD-nanobody/FT-GO 3D-IHC strategy, which combines peroxidase-fused nanobodies with tyramide-glucose oxidase amplification, enables rapid, deep, and ultrasensitive labeling in millimetre-thick tissues (Yamauchi et al., 2024). These innovations also catalyzed a new wave of integrative platforms. For instance, DISCO-MS (3D imaging of solvent-cleared organs profiled by mass spectrometry) combined vDISCO with deep-learning-based image analysis, robotic tissue extraction, and ultrahigh-sensitivity mass spectrometry allowing researchers to isolate minute fluorescently labeled regions from intact mouse or human organs and probe their molecular composition with unprecedented precision (Bhatia et al., 2022).

The other labeling toolkit for TC remains using smaller, faster diffusing probes, such as antibody fragment-antigen binding (Fabs, 50 kDa) units, antibody single-chain Fv fragments (scFv, 25 kDa), and nucleic acid-based aptamers, hold theoretical advantages in deep tissue penetration and rapid labeling (Condado-Morales et al., 2024; Crivianu-Gaita and Thompson, M, 2016). Some studies have explored 3D cleared tissues including SHIELD (Stabilization under Harsh Conditions via Intramolecular Epoxide Linkages to prevent Degradation) (Park et al., 2019), PACT (Treweek et al., 2015). Notably, the development of thermostable Fab-complexed antibodies has further enhanced deep penetration while reducing the number of antibodies required (Lai et al., 2022).

On the other hand, multiround staining has been shown to be successfully compatible with several TC methods (e.g., CLARITY, SHIELD, SWITCH (System-Wide control of Interaction Time and kinetics of CHemicals), SHARD, HIF-Clear, MOCAT (Multiplex labeling Of Centimeter-sized Archived Tissue), SHORT (SWITCH-H_2_O_2_-antigen Retrieval-TDE (Chung et al., 2013; Murray et al., 2015; Park et al., 2019; Lin et al., 2024; Pesce et al., 2022; Rosen et al., 2024). Building on these progresses, recent technical innovations have extended the applicability of multiround staining to developing and diseased tissues, human CLARITY samples, and zebrafish neural circuit mapping. In particular, the combination of tyramide signal amplification with PACT (TSA-PACT) enables subsequent restaining of the same tissue section and single-resolution imaging, providing researchers with richer molecular information and facilitating more comprehensive biological interpretation (Wang, et al., 2023; Woelfle et al., 2023).

Despite its widespread use, immunolabeling remains constrained by limited tissue penetration, incomplete labeling uniformity, and sensitivity to clearing conditions, prompting sustained efforts to refine existing techniques and explore alternative molecular labeling strategies.

##### 2.1.2.2 Chemically mediated labeling

Beyond immunolabeling, recent advances in bioorthogonal reactions have expanded the scope of TC imaging toward molecular interrogation. Click chemistry-based labeling represents an important class of these strategies, which exploit bioorthogonal chemical reactions to achieve rapid and highly specific molecular labeling. Representative approaches include *in situ* Cu-catalyzed azide-alkyne cycloaddition (CuAAC), which has been widely applied for 3D visualization of covalent drugs and DNA synthesis markers (Lazutkin et al., 2019; Pang et al., 2022). More recently, the developed Click3D method improved staining depth and enables high-resolution visualization of nascent RNA and other molecular targets across intact organs (Tamura et al., 2024). Additionally, metabolic labeling strategies have also been integrated with TC imaging, where a fluorescent D-amino acids (FDAA) are incorporated into bacterial peptidoglycan, enabling 3D visualization of indigenous gut microbiota within intact tissues (Wang et al., 2021a; Wang et al., 2024).

##### 2.1.2.3 Nucleic-acid-based labeling

Complementing protein-targeted approaches, mRNA labeling strategies have increasingly been integrated with TC workflows, enabling spatial visualization of gene expression in intact tissues. Representative methods include RNA fluorescence *in situ* hybridization (RNA-FISH) and its derivatives, such as HCR-FISH (Kumar et al., 2021) and EASI-FISH (Wang et al., 2021b), which permit highly sensitive detection of transcripts in cleared specimens. More recently, advances in spatial transcriptomic technologies, including STARmap (Wang et al., 2018), MERFISH (Zhang et al., 2021a), and related spatial transcriptomic approaches, have further expanded the capacity for 3D mapping of gene expression patterns. Although challenges related to signal retention, probe penetration, and multiplexing remain, these approaches represent a promising direction for integrating transcriptomic information with volumetric tissue imaging.

#### 2.1.3 Functional labeling

Another emerging strategy involves activity-based covalent probes, which enable the visualization of specific enzymatic activities within intact tissues. For instance, ANA-o-BODIPY, an enzymatically activatable probe, allows whole-organ imaging of aminopeptidase N (APN) activity, capturing spatial heterogeneity and responses to inhibitors (Yi et al., 2025). In parallel, there are microenvironment-responsive probes that typically rely on oxygen-sensitive activation mechanisms, allowing visualization of local physiological states such as oxygen tension in whole-organ and whole-body imaging at single-cell resolution (Sakamoto et al., 2024). These functional labeling strategies are typically enabled by activatable covalent probes, which generate reactive intermediates upon target activation and form irreversible bonds with nearby biomolecules, thereby ensuring signal retention during TC.

These innovations collectively highlight a progressive transition from structural visualization to molecular specificity and functional interrogation, enabling comprehensive, multiscale mapping of biological systems in 3D tissues.

### 2.2 Genetically encoded labeling

Genetically encoded labeling strategies enable the direct incorporation or covalent attachment of fluorescent reporters to proteins of interest (POIs), thereby facilitating precise visualization and tracking of molecular interactions and cellular dynamics (Figure 2). In addition to transgenic approaches, viral vectors are widely used to deliver genetically encoded reporters into specific tissues and cell populations, thereby extending the versatility and experimental applicability of genetically encoded labeling.

#### 2.2.1 Direct fluorescent reporters

Fluorescent proteins (FPs) typically ranging from 25 to 35 kDa in size and approximately 4–5 nm in diameter, such as green fluorescent protein (GFP), red fluorescent protein (RFP), and mCherry, are genetically encoded reporters that can be expressed in specific cell populations or organ systems, enabling visualization of molecular interactions within their native cellular context (Chudakov et al., 2010; Laxman et al., 2021; Streit et al., 2025). The continuous evolution of FPs has yielded variants with improved brightness, photostability, and spectral diversity, facilitating efficient and long-term imaging in intact tissues (Deliolanis et al., 2014). Among the various TC protocols, several have demonstrated excellent fluorescence preservation when applied to reporter animals such a*s Thy1-YFP-H* mice, *Thy1-GFP-M* mice, *R26R-tdTomato* mice, as well as conditional reporter models generated through the Cre-LoxP recombination system, including *Tie2-Cre;Ai14* and *Cdh5-CreERT2* mice (Jing et al., 2018; Biswas et al., 2021). In particular, methods including CLARITY (Chung et al., 2013), FOCM (Zhu et al., 2019), PEGASOS (Jing et al., 2018), Ce3D (Li et al., 2019), CUBIC (Susaki et al., 2014), Fast 3D Clear (Kosmidis et al., 2021), SHIELD (Stabilization to harsh conditions via intramolecular epoxide linkages to prevent degradation) (Park et al., 2019), and SOLID (Suppressing tissue distortion based on synchronized dehydration/delipidation treatment with 1,2 hexanediol [1,2-HxD] mixtures) (Zhu et al., 2024) have been reported to exhibit superior fluorescence retention and structural integrity of fluorescence. These active labeling strategies offer stable, cell-type-specific fluorescence signals for TC-based volumetric imaging without the need for additional staining. However, their application is limited to specific transgenic strains and animal models, making them unsuitable for human specimens, and not all TC methods are fully compatible with stable fluorescent protein preservation. This approach also faces challenges such as limited spectral flexibility and relatively weak signal emission.

#### 2.2.2 Genetically encoded chemical labeling systems

Self-labelling enzyme tags, exemplified by Halo-, SNAP-, CLIP-, and TMP-tags, with a molecular size of approximately 20–30 kDa, are engineered enzyme-based labelling systems that covalently react with specific synthetic substrates (Laxman P. et al., 2021). These systems provide greatly enhanced brightness, photostability, and chemical tunability compared with FPs (Wilhelm et al., 2021; Porzberg et al., 2025). Pioneering work in 2014 first demonstrated that self-labeling protein tags for rapid, uniform, and low-background labeling in thick *Drosophila* and mouse brain tissues (Kohl et al., 2014), and subsequent Tetbow-based multicolor studies confirmed their compatibility with solvent-based clearing methods such as 3DISCO and BABB, where FP signals are strongly quenched (Sakaguchi et al., 2018).

On the other hand, short peptide tags (0.6–6 kDa) have emerged as an alternative strategy that introduces minimal steric perturbation to the fused protein while serving as substrates for highly specific enzyme-catalyzed labeling reactions. Representative examples include the His-, FLAG-, HAT-, and HA-tag, which enable selective labeling of the POIs with synthetic ligands (Uchinomiya et al., 2014; Lotze et al., 2016). More recently, the SPARKLE (Strategic Peptide, Anchored, Retained, and Kept after Lipid Elimination) system, further demonstrated that rationally designed peptides can support stable retention and high-resolution labeling of nanoparticles compatible with CLARITY clearing (Balcorta et al., 2025). Given their small molecular weights, efficient tissue penetration, high penetrance, and covalent stability under harsh clearing conditions, these chemical tags may become increasingly valuable for next-generation site-specific labeling, particularly as efforts continue to enhance their compatibility with diverse TC protocols.

#### 2.2.3 Enzyme-mediated labeling

An engineered, solubility-enhanced TurboID (seTurboID) biotin ligase enables efficient *in vivo* proximity biotinylation. Subsequent whole-mount staining with monovalent streptavidin-conjugated fluorophores allows rapid, low-background, and highly penetrant labeling, providing a versatile alternative for high-contrast molecular visualization in large, optically cleared specimens (Zhong et al., 2025).

### 2.3 Label-free imaging

Beyond exogenous and genetic labeling, label-free imaging approaches leverage intrinsic tissue properties, such as autofluorescence, nonlinear optical responses, RI contrasts, optical absorption, and scattering, to extract structural and biochemical information without the need for external probes or molecular labels. In addition, label-free imaging often provides improved imaging depth and reduced perturbation, making it particularly advantageous for large-scale, intact tissue imaging when combined with TC (Figure 2).

#### 2.3.1 Autofluorescence signal

Autofluorescence originating from endogenous molecules such as NADH, collagen, elastin, and lipofuscin, can decrease the signal-to-noise ratio (SNR) and reduce image contrast during fluorescence imaging. Its broad emission spectrum (approximately 400–700 nm) often overlaps with commonly used fluorescent dyes, leading to spectral cross-talk. Consequently, most TC protocols aim to quench autofluorescence, thereby improving imaging fidelity (Croce and Bottiroli, 2017; Richardson et al., 2021). Nevertheless, autofluorescence can also be exploited as an intrinsic contrast for label-free imaging, enabling visualization of tissue architecture and pathological alterations without exogenous labeling. For instance, using light-sheet or two-photon microscopy combined with TC, studies have visualized stiffness-related collagen alterations in the cornea (Stoecker et al., 2024), characterized tissue-biomaterial interactions at subcellular resolution (Ngo et al., 2023), and reconstructed the 3D architecture of the enteric nervous system (Hazart et al., 2023).

#### 2.3.2 Nonlinear optical (NLO)-based contrast

NLO microscopy represents an important category of intrinsic contrast imaging, encompassing multiple modalities based on distinct physical mechanisms. For instance, second harmonic generation (SHG), stimulated Raman scattering (SRS) microscopy, and multiphoton microscopy (MPM) have been successfully applied to skeletal muscle, tumour and brain tissues, enabling high-resolution assessment of extracellular matrix, cellular architecture, and metabolic activities without exogenous labels (Wei et al., 2019; Schneidereit et al., 2021; Touloumes et al., 2021; Pichon et al., 2022; Makki et al., 2025). Notably, Raman dyes combined with a dedicated TC strategy (rDISCO) and SRS, enable multiplexed imaging of multiple targets in thick samples within a single acquisition, while extending imaging depth by 10–100 fold compared to previous methods (Shi et al., 2022).

#### 2.3.3 RI-dependent optical contrast

RI-dependent contrast represents another important mechanism for label-free imaging. In cleared tissues, RI homogenization reduces scattering while preserving subtle spatial variations in optical density, which can be exploited to distinguish structural components. It has been applied in the FxClear protocol to visualize white and grey matter in cleared brain tissues, using both conventional optical microscopy and optical coherence tomography (OCT) (Lee et al., 2021).

#### 2.3.4 Absorption-based intrinsic contrast

Photoacoustic imaging represents a key modality primarily by exploiting endogenous optical absorption of biomolecules. Ultraviolet photoacoustic microscopy (UV-PAM) combined with TC enhances contrast and depth for whole-organ imaging. Similarly, TC-assisted photoacoustic imaging (PAI) enables label-free, high-resolution, and deep 3D mapping of vascular and lymphatic networks *in vivo* (Novoselova et al., 2020; Li et al., 2022).

#### 2.3.5 Scattering-based contrast

By leveraging intrinsic backscattering signals, the combination of TC with optical frequency domain imaging (OFDI) and OCT enables intact brains to be imaged at optical resolution without labeling, allowing deep-tissue structural visualization based on endogenous scattering properties (Ren et al., 2017).

Label-free imaging in TC offers a versatile, complementary framework for 3D structural, chemical, and functional analysis. Notably, while these methods enable visualisation of macroscopic structures, their molecular-level specificity remains limited, making the distinction of individual proteins, receptors, or low-abundance biomolecules challenging.

## 3 Cross-cutting techniques for improving labeling efficiency

Although numerous labeling strategies have emerged, immunolabeling and FPs remain the most widely employed and broadly compatible approaches in TC (Richardson et al., 2021). Conventional approaches typically enhance labeling efficiency by reducing sample size or by serial sectioning, followed by reconstruction of 2D sections into 3D tissue structures (Richardson et al., 2021; Kiemen et al., 2023). Recent methodological improvements have increasingly focused on enhancing antibody penetration, promoting labeling homogeneity, and preserving intrinsic FP fluorescence.

### 3.1 Chemical improved labeling strategies

**FIGURE 3:**
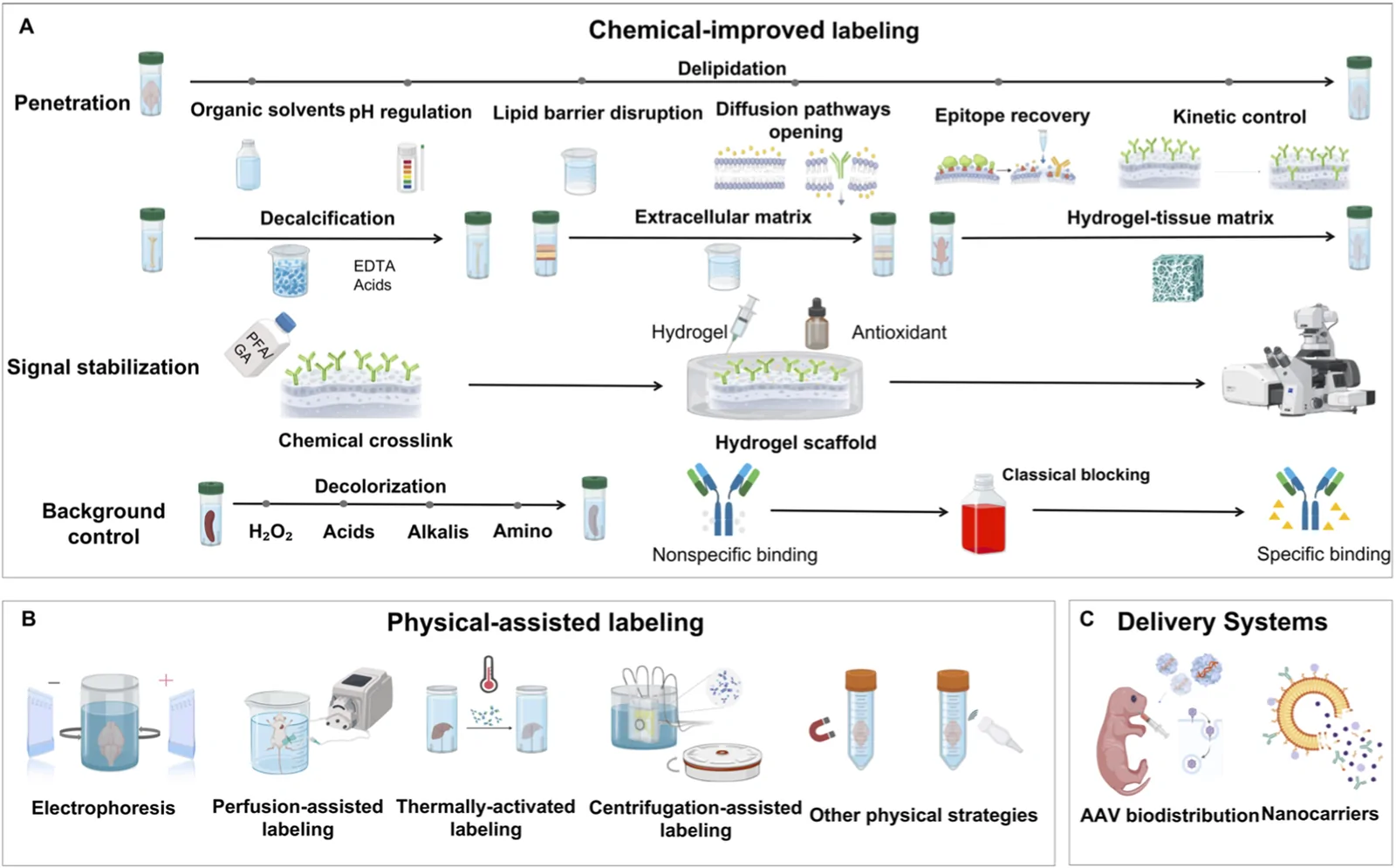
Strategies to enhance labeling efficiency in tissue clearing (TC)-based volumetric imaging. **(A)** Chemical approaches for improving molecular penetration, signal preservation, and background control. Delipidation using organic solvents, pH modulation, and detergents disrupts lipid barriers, increases tissue permeability, and facilitates epitope exposure and diffusion kinetics. Decalcification (e.g., EDTA and acidic treatments) improves probe accessibility in mineralized tissues. ECM modulation enhances probe penetration by alleviating matrix-associated diffusion barriers. Hydrogel-tissue hybirds shorten the diffusion distance and increase the pore size. Signal stabilization is achieved through chemical crosslinking (e.g., PFA, GA) and hydrogel embedding, which preserve protein structures and fluorophore anchoring during prolonged clearing and imaging. Background reduction strategies include chemical decolorization (e.g., H_2_O_2_, acids, alkalis, amino reagents) to suppress endogenous pigments, along with classical blocking reagents to minimize nonspecific antibody binding and enhance labeling specificity. **(B)** Physical-assisted labeling strategies. External physical forces are employed to accelerate probe delivery and improve tissue penetration, including electrophoresis-driven transport, perfusion-assisted labeling, thermally activated diffusion, centrifugation-assisted infiltration, as well as emerging approaches such as magnetic force or magnetohydrodynamic (MHD)-mediated focusing and low-frequency ultrasound (LFU)-assisted delivery. **(C)** Delivery systems supporting labeling strategies. Advanced delivery platforms, including viral vectors (e.g., AAV for biodistribution and cell type-specific labelling) and nanocarriers, enable efficient, targeted, and scalable probe transport within cleared tissues, further expanding the versatility of TC-compatible labeling approaches. Illustration created with BioRender. AAV: adeno-associated viruse; ECM: extracellular matrix; EDTA: ethylenediaminetetraacetic acid; GA: glutaraldehyde; PFA:paraformaldehyde.

In TC workflows, chemical strategies to improve labeling primarily focus on enhancing molecular permeability, signal stability, and reducing intrinsic tissue background, thereby optimizing the chemical microenvironment to increase the accessibility, uniformity, and specificity of macromolecular probes (Figure 3A).

#### 3.1.1 Penetration improvement

Improving the penetration of external molecules remains a priority, especially for dense, lipid-rich tissues, because there is a high RI in most biological tissues. Although organic solvents (e.g., alcohols, tetrahydrofuran) enable rapid delipidation, they often cause morphological distortion and quench FPs (Richardson and Lichtman, 2015; Yu et al., 2021). Several refinements, for example, increasing the number of steps in the gradient, adjusting alkaline pH (Li et al., 2018; Qi et al., 2019), incubating THF with dibenzyl ether (DBE) or dichloromethane (DCM) (Ertürk et al., 2012; Mai et al., 2022), and applying dehydration/rehydration cycles in iDISCO variants, help mitigate these drawbacks. More recently, by using cholesterol extraction to open diffusion pathways (in the wildDISCO workflow), allowing full-length IgG antibodies to penetrate tissues up to 2 cm thick has advanced permeabilization (Mai et al., 2024). Detergent-based delipidation (e.g., Sodium dodecyl sulfate (SDS), Triton X-100, CHAPS (3-[(3-cholamidopropyl) dimethylammonio]-1-propanesulfonate)) remains a critical strategy in methods such as CLARITY, or sodium cholate (SC) and a zwitterionic, etc., loosening lipid barriers and promoting diffusion (Na et al., 2022; Baek et al., 2024; Yu, et al., 2017). Similarly, aminoalcohols such as Quadrol (Matsumoto et al., 2019) and alkaline solution (AKS) (Shan et al., 2022), also play a key role in lipid removal.

Notably, some delipidation buffers simultaneously facilitate epitope recovery. For example, FLASH employs zwittergent and urea to unmask epitopes and improve permeability for high-molecular-weight probes in dense tissues (Messal et al., 2021), while MOCAT, SHARD extend similar advantages to FFPE samples, enabling 3D reconstruction (Lin et al., 2024; Rosen et al., 2024). In addition, extensive research has been done on kinetic control strategies to facilitate deep immunolabeling, including system-wide reaction inactivation and subsequent reactivation (SWITCH) (Murray et al., 2015), partial reaction inhibition via specialized buffer formulation (CUBIC-HistoVision) (Woelfle et al., 2023), and some overall platforms (SHIELD, SHORT) (Park et al., 2019; Pesce et al., 2022). Nevertheless, despite these advances, their effectiveness has not yet been fully validated across different tissue types and specimen scales. Achieving consistently homogeneous labeling in large and dense tissues remains an ongoing challenge.

Beyond delipidation, tissue preparation often requires region-specific decalcification to optimize labeling efficiency and imaging quality. Decalcification, necessary for mineralized tissues like bone and teeth, facilitates probe penetration and optical clearing, with agents such as ethylenediaminetetraacetic acid (EDTA) (e.g., CUBIC-Bone, PEGASOS, Bone-mPACT+,.etc.), nitric acid, formic acid, or hydrochloric acid (HCl) applied in aqueous or buffered solutions according to tissue thickness and mineral content (Jing et al., 2019; Jin et al., 2023; Yu et al., 2017).

In addition, extracellular matrix (ECM)-associated diffusion barriers can also limit probe penetration, particularly in dense connective tissues, fibrotic organs, and large human specimens. Recent studies have explored ECM-modulating strategies to improve molecular accessibility. For example, the SHANEL protocol introduced CHAPS and N-methyldiethanolamine to facilitate antibody penetration and labeling in centimeter-scale human tissues (Mai et al., 2022). In parallel, decellularization-based approaches have enabled comprehensive 3D mapping of native ECM proteins across multiple organs by removing cellular components while preserving the underlying matrix architecture (Mayorca-Guiliani et al., 2019). These strategies may be particularly valuable for imaging ECM-rich tissues such as skin, tendons, ligaments, and fibrotic tissues.

Specifically, in hydrogel-based TC methods, chemical swelling of the hydrogel-tissue matrix has been shown to enhance probe penetration. For instance, the novel technique ELAST (Entangled Link-Augmented Stretchable Tissue) converts tissues into elastic hydrogel-tissue hybrids that can be macroscopically deformed, thereby shortening diffusion distances and improving probe infiltration (Ku et al., 2020). Similarly, incubation of cleared samples in a boric acid buffer during washing and labeling transiently expands the hydrogel network, increasing pore size and facilitating deep antibody diffusion (Du et al., 2019).

#### 3.1.2 Signal stabilization

Stabilizing signals during extended clearing and imaging represents another critical determinant of labeling performance**.** Long clearing durations, repeated solvent exposure, and high-intensity light-sheet imaging can progressively degrade protein-bound fluorophores and compromise antibody integrity. Chemical crosslinkers such as paraformaldehyde (PFA) and glutaraldehyde (GA) help retain protein scaffolds and preserve fluorophore anchors throughout processing, whereas the introduction of hydrogel-forming acrylamide monomers marked a milestone that substantially enhanced labeling uniformity and imaging outcomes (Gradinaru et al., 2018; Richardson et al., 2021). CLARITY and its derivatives, or hybrid cocktail approaches, demonstrate clear advantages in fluorophore preservation and antibody penetration homogeneity across species and across different clearing methodologies (Guo et al., 2022; Jin et al., 2023). Meanwhile, antioxidant and anti-fade reagents (e.g., vitamin E; N,N,N′,N′-tetrakis (2-hydroxypropyl)ethylenediamine (EDTP) used in FDISCO+) are increasingly incorporated into staining buffers and RI matching solutions to suppress photo-oxidation and sustain fluorescence intensity during prolonged imaging sessions (Wan et al., 2021; Feng et al., 2024).

#### 3.1.3 Background control

Reducing background and autofluorescence has become a critical requirement for high-resolution, deep-tissue imaging, due to light absorbing pigments, including haemoglobin, melanin, and lipofuscin, accumulate in tissues and generate strong autofluorescence signals (Molbay et al., 2021). Consequently, decolorization is particularly important for heavily pigmented organs such as the liver, spleen, and skin, and is commonly achieved using hydrogen peroxide (H_2_O_2_), strong acids, or strong alkalis. Recent advances have further introduced amino-based chemical quenching strategies (e.g., NH_4_OH), which complement solvent-clearing workflows and offer an additional route for reducing background across different clearing modalities (Tainaka et al., 2016; Richardson et al., 2021; Yu et al., 2021). Moreover, classical blocking reagents such as Bovine serum albumin (BSA), gelatin, and serum remain essential to minimize nonspecific antibody adsorption and constitute essential preparatory steps across multiple TC systems (Renier et al., 2014; Richardson et al., 2021; Tian et al., 2021). However, their effectiveness may be context-dependent, and recent studies have suggested that certain blocking reagents, particularly BSA, may not always improve signal quality and can even reduce fluorescence performance in thick optically cleared tissues (Chwastowicz et al., 2025).

### 3.2 Physical-assisted labeling strategies

#### 3.2.1 Electrophoresis

Electrophoretic clearing and labeling, Chung et al. (2013) introduced active transport organ-electrophoresis in CLARITY, where an electric field accelerates delipidation mediated by ionic detergents. Despite its efficiency, this approach is difficult to implement and prone to tissue damage from heating and epitope loss. Subsequent refinements, including stochastic electrotransport and ACT (active clarity technique), use gentler or rotational electric fields to enhance probe penetration, or incorporate temperature-control modules, while reducing protein loss and heat induced injury, and remain compatible with other chemical clearing methods (Kim et al., 2015; Lee et al., 2016; Yun et al., 2025). With further development, advanced platforms, like PRE-CLARITY, adopting a non-circulation electrophoresis system (Du et al., 2019), EERS (electro-enhanced rapid staining) enable rapid and efficient immunostaining of targeted tissues (Zhang et al., 2021b) (Figure 3B; Table 1).

**TABLE 1:** Physical assisted labeling strategies.

Labeling type	Strategies	TC protocal	Imaging scale	Tissue volume	Molecular targets	Additional notes	References
Electrophoretic-labeling	Hydrogel fusion + active transport electrophoresis	CLARITY	Whole-organ imaging	Adult mouse/human brain	Antibodies, small-molecules, fluorescence signal	Multi-round molecular phenotyping	Chung et al. (2013)
	Electrophoretically driven	Stochastic electrotransport + CLARITY	Whole-organ imaging	Mouse multiple organ	Antibodies, small-molecules, fluorescence signal	Compatibility with other chemical clearing methods	Kim et al. (2015)
	Electrophoretically driven	eFLASH	Whole-organ imaging	Mouse, human-IPSC, marmoset	Antibodies, small-molecules	​	Yun et al. (2025)
	NCES	PRE-CLARITY	Whole-organ imaging	Mouse brain	Antibodies, small-molecule	​	Du et al. (2019)
	Electrophoretically driven	EERS + CLARITY	Slices	Adult mouse/human brain	Antibodies	​	Zhang et al. (2021)
Perfusion-assisted labeling	Perfusion-assisted agent release *in situ*	PARS	Whole-organ/whole body imaging	Mice	Antibodies, small-molecules, mRNA probes, fluorescence signal	​	Yang et al. (2014)
	Pressure driving (ETC., chamber system)	ACT-PRESTO	Whole-organ/whole body imaging/slices	Rodents	Antibodies, small-molecules, fluorescence signal	Compatibility with other chemical clearing methods	Lee et al. (2016)
	Pressure driving	vDISCO	Organ/whole body imaging	Mice	Nanobodies, fluorescence signal	​	Cai et al. (2023)
	Pressure driving	SHANEL	Whole-organ	Human organs	Antibodies, small-molecules	​	Mai et al. (2022)
Thermally activated labeling	Thermal driven	PACT	Whole-organ imaging	Mouse brain	Antibodies, fluorescence signa	​	Yu et al. (2017)
​	Thermal driven	SPEARs + ThICK staining	Whole-organ/whole tissue imaging	Mouse/human brain	Antibodies	​	Lai et al. (2022)
Centrifugation-assisted labeling	Centrifugation driven	c-PRESTO	Slices	Thick brain (rodents)	Antibodies	ACT-PRESTO 600 rcf	Lee et al. (2016)
​	Centrifugation driven	CEx staining	Whole-organ imaging	Mouse brain	Antibodies	PRE-CLARITY rotation speed to 1,200 r/min	Du et al. (2019)
Other physical enhancement strategies	Magnetic force	Magnetic force + CLARITY	Whole-organ	Alzheimer’s disease post-mortem brain samples	Antibodies, small-molecules	​	Na et al. (2021)
	Magnetohydrodynamic force	MHD-accelerated clearing	Whole-organ	Mouse and zebrafish brain/adult nudibranch	Antibodies, fluorescence signal	​	Dwyer et al. (2021)
​	Low frequency ultrasound	SoniC/S	Whole-organ/tissue	Rat	Antibodies	Compatible with iDISCO/PEGASOS	Cheung et al. (2025)

CEx: Centrifugation expansion; c-PRESTO: centrifugal-pressure related efficient and stable transfer of macromolecules into organs eFLASH: electrophoretically driven fast labeling using affinity sweeping in hydrogel; EERS: electro-enhanced rapid staining; ETC.,: Electrophoretic tissue clearing; iDISCO: immunolabeling-enabled 3D imaging of solvent-cleared organs; IPSC: Induced pluripotent stem cell; MHD: magnetohydrody; NCES: Non-circulation electrophoresis system; PEGASOS: Polyethylene glycol (PEG)-associated solvent system; PACT: Passive CLARITY, technique; PARS: Perfusion-assisted agent release *in situ*; rcf: Relative centrifugal force; SoniC/S: sonication ssisted tissue clearing and immunofluorescent staining method; ThICK: hermo-immunohistochemistry with optimized kinetics; vDISCO: nanobody (VHH)-boosted 3D imaging of solvent-cleared organs; SHANEL: Small-micelle-mediated human organ efficient clearing and labeling; SPEARs: Synergistically protected polyepoxide-crosslinked Fab-complexed antibody reagents.

#### 3.2.2 Perfusion-assisted labeling

Another effective strategy is perfusion-assisted labeling. The PARS method (Perfusion-Assisted Agent Release *in Situ*) leverages the intact vasculature of the animal to deliver hydrogel monomers and clearing solutions directly, allowing them to diffuse uniformly throughout the tissues of interest (Treweek et al., 2015; Yang et al., 2014). Moreover, vDISCO combines pressure-driven perfusion with nanobody-based labeling, enhancing fluorescent protein signals by up to two orders of magnitude (Cai et al., 2019). Other approaches, including PRESTO (Pressure-Related Efficient and Stable Transfer of Macromolecules into Organs) (Lee et al., 2016) and SHANEL (Mai et al., 2022), also use pressure-driven clearing strategies to achieve efficient labeling of antibodies and small molecules in animal and human tissues, enabling comprehensive whole-organ imaging and visualization (Figure 3B; Table 1).

#### 3.2.3 Thermally-activated labeling

This method enhancement of molecular diffusion has emerged as an effective strategy to accelerate antibody or probe penetration in deep tissue (Tainaka et al., 2016). Studies indicate that moderate elevation of temperature, such as 42 °C–47 °C is an alternative temperature range for PACT, can significantly increase solute diffusivity within the tissue matrix, thereby speeding up labeling and clearing processes, while careful optimization is required to balance the trade-off between improved penetration and potential drawbacks such as structural damage, protein denaturation, or fluorescence quenching (Lai et al., 2022; Yu et al., 2017) (Figure 3B; Table 1).

#### 3.2.4 Centrifugation-assisted labeling

In addition, several studies have employed centrifugal force to accelerate molecular labeling. For example, Lee et al. (2016) demonstrated that c-PRESTO (centrifugal-PRESTO) markedly enhances antibody penetration in thick brain slices by centrifuging samples at 600 × G for 3 h. Building on this concept (Du et al., 2019), further developed Centrifugation-Expansion (CEx) staining, in which higher rotation speeds and prolonged centrifugation enabled effective immunolabeling of intact mouse brains (Figure 3B; Table 1).

#### 3.2.5 Other physical enhancement strategies

In addition to conventional transport mechanisms, several emerging physical approaches have been developed further to improve molecular delivery and labeling efficiency in cleared tissues. For instance, magnetic force (Dwyer et al., 2021) or magnetohydrodynamic (MHD) force (Na et al., 2021) mediated electric field focusing can enhance convective transport, while low-frequency ultrasound (LFU)-assisted sonication integrated with organic-solvent-based TC (Cheung et al., 2025), facilitates antibody penetration by increasing tissue permeability (Figure 3B; Table 1).

### 3.3 Delivery systems supporting labeling strategies

Recombinant adeno-associated viruses (rAAVs), have emerged as versatile tools for both *in vitro* and *in vivo* gene delivery. *Cre* recombination-based AAV targeted evolution (CREATE), which achieves highly efficient and cell-type-specific transduction, including widespread transduction of the adult central nervous system (Deverman et al., 2016). When combined with TC and volumetric imaging allows whole-organ, high-resolution visualization of AAV biodistribution, neural circuit tracing, and single-cell morphological analysis while preserving endogenous fluorescence (Kosmidis et al., 2021; Lopes et al., 2022; Zhu et al., 2024). Moreover, gene therapy strategies employing localized, *in situ* production of therapeutic proteins, such as the SHielded, REtargeted ADenovirus (SHREAD) platform, demonstrate enhanced efficacy and reduced systemic toxicity, with PACT techniques facilitating precise 3D quantification of local transgene expression (Smith et al., 2021) (Figure 3C).

Nanocarriers provide a versatile platform for interfacing with biological systems at the molecular scale, thereby enhancing imaging contrast and enabling functional interrogation of tissues. A broad range of nanomaterials, including lipid-based nanoparticles, polymeric nanoparticles, and metal-based nanostructures, etc., have demonstrated considerable success in TC applications, as reviewed in detail elsewhere (Rong et al., 2025). Recent methodological advances have further expanded these capabilities. For example, the SCP-Nano (Single Cell Precision Nanocarrier Identification) framework enables organism-wide, single-cell-resolution mapping of nanocarrier biodistribution by integrating whole-body clearing and labeling strategies with deep learning analysis, achieving detection at doses as low as 0.0005 mg kg^-1^ and thus providing a 100–1,000 fold increase in sensitivity over conventional approaches while generalizing across diverse nanocarrier platforms (Luo et al., 2025) (Figure 3C).

In summary, such delivery systems could enhance labeling efficiency and imaging quality in TC workflows by improving molecular penetration, increasing cell-type specificity, preserving high SNR, and enabling functional labeling across diverse tissue contexts.

## 4 Conclusion and perspectives

The application of TC has progressively evolved towards multiomics approaches, integrating artificial intelligence and other methodologies to elucidate the 3D spatial conformation of complex living organisms. Progresses in labeling strategies have substantially expanded the capabilities of TC and volumetric imaging.

Beyond traditional immunolabeling and fluorescent proteins, a new generation of chemical probes*,* engineered biosensors*,* and label-free approaches has accelerated progress by enabling more versatile and fine-tuned molecular targeting. Meanwhile, labeling capacity has also advanced from single target detection to iterative and multiround staining, providing a practical route to multiplexed molecular profiling in large, cleared tissues. At the same time, the rapid adoption of nanobodies has further broadened the scope of *in situ* interrogation, allowing researchers to visualize cellular structures, proteins, and nucleic acids with single-cell resolution. Although compact binders such as Fabs, scFvs, and aptamers offer clear advantages in molecular size and engineering flexibility, research on their use in large-scale TC remains limited, and their broader implementation will require substantial further development and rigorous validation, particularly in terms of stability, penetration, and batch-to-batch reproducibility. Notably, recent approaches integrating genetic code expansion (GCE) driven *in situ* click labeling have enabled rapid imaging-based selection of functional nanobody dye conjugates. Although such strategies have not yet been widely applied to large-volume TC contexts, they represent a promising direction for future developments by combining advanced labeling chemistry with high-throughput imaging capabilities (Seike et al., 2025).

On the other hand, chemical enhancement of labeling efficiency remains a central component across nearly all clearing platforms. Numerous groups and clearing frameworks have introduced impactful innovations that continue to drive rapid progress in large-volume imaging. Contributions from physical-assisted methods that transform passive diffusion into active delivery, as well as carrier-based targeting systems, have markedly improved labeling homogeneity, specificity, and depth. Importantly, many clearing techniques use integrated workflows rather than single-component processes, such as CLARITY combines hydrogel embedding with electrophoretic TC, vDISCO integrates nanobody labeling with active perfusion, and a trio of PACT, PARS and RIMS offered a user-friendly, rapid approach to rendering whole organs and whole organisms transparent. Similarly, strategies to enhance penetration or preserve fluorescence often rely on carefully tuned chemical cocktails, and methods such as SHORT support cross-modal coordination between solvent-based, aqueous, and hybrid systems. These modular elements enable researchers to tailor clearing and labeling conditions to the specific biological features and experimental goals of each study. To date, no single labeling strategy is universally optimal for all TC applications. Selecting an appropriate labeling strategy requires balancing specificity, penetration depth, multiplexing capability, and experimental feasibility according to the biological question of interest.

However, due to space limitations, many excellent TC methods and applications could not be discussed in this review. We have therefore focused on representative milestones and recent advances, with particular emphasis on labeling strategies that can be implemented before or after the *ex vivo* clearing process. Improvements that are tightly coupled to specific high-resolution microscopy platforms can be found in dedicated reviews elsewhere. Notably, recent developments, such as the bovine serum albumin-based clearing medium (SeeDB-Live), which is compatible with living cells, begin to challenge the long-standing limitation of TC-associated cytotoxicity and open the possibility for live-tissue clearing and imaging (Inagaki et al., 2026). This emerging direction is expected to fundamentally expand the scope of TC, paving the way toward dynamic and functional interrogation of biological processes in living systems.

## References

[B1] Almagro J. Messal H. A. Zaw Thin M. Van Rheenen J. Behrens A. (2021). Tissue clearing to examine tumour complexity in three dimensions. Nat. Rev. Cancer 21, 718–730. 10.1038/s41568-021-00382-w 34331034

[B2] Baek S. Jang J. Jung H. J. Lee H. Choe Y. (2024). Advanced immunolabeling method for optical volumetric imaging reveals dystrophic neurites of dopaminergic neurons in alzheimer’s disease mouse brain. Mol. Neurobiol. 61, 3976–3999. 10.1007/s12035-023-03823-9 38049707 PMC11236860

[B3] Balcorta H. V. Corral M. Y. M. Gallegos A. Chavez J. Perez J. Balivada S. (2025). Development of chemical tags for universal lipid nanoparticle visualization and tracking in 2D and 3D imaging. Nano Lett. 25 (19), 7682–7689. 10.1021/acs.nanolett.5c00311 40319395 PMC12082698

[B4] Bhatia H. S. Brunner A.-D. Öztürk F. Kapoor S. Rong Z. Mai H. (2022). Spatial proteomics in three-dimensional intact specimens. Cell. 185, 5040–5058.e19. 10.1016/j.cell.2022.11.021 36563667

[B5] Biswas L. Chen J. De Angelis J. Chatzis A. Nanchahal J. Dustin M. L. (2021). SUMIC: A Simple Ultrafast Multicolor Immunolabelling and Clearing Approach for Whole-Organ and Large Tissue 3D Imaging. 10.1101/2021.01.20.427385

[B6] Blain R. Couly G. Shotar E. Blévinal J. Toupin M. Favre A. (2023). A tridimensional atlas of the developing human head. Cell. 186, 5910–5924.e17. 10.1016/j.cell.2023.11.013 38070509 PMC10783631

[B7] Cai R. Pan C. Ghasemigharagoz A. Todorov M. I. Förstera B. Zhao S. (2019). Panoptic imaging of transparent mice reveals whole-body neuronal projections and skull–meninges connections. Nat. Neurosci. 22, 317–327. 10.1038/s41593-018-0301-3 30598527 PMC6494982

[B8] Cai R. Kolabas Z. I. Pan C. Mai H. Zhao S. Kaltenecker D. (2023). Whole-mouse clearing and imaging at the cellular level with vDISCO. Nat. Protoc. 18, 1197–1242. 10.1038/s41596-022-00788-2 36697871

[B9] Chen Z. Ji S. Huang C. Tu W. Ren X. Guo R. (2024). *In situ* reprogramming of cardiac fibroblasts into cardiomyocytes in mouse heart with chemicals. Acta Pharmacol. Sin. 45, 2290–2299. 10.1038/s41401-024-01308-6 38890526 PMC11489685

[B10] Cheung H. P. H. Lauwers M. Wang Z. Wang J. Ning C. Ker D. F. E. (2025). Rapid sonication-assisted whole tissue clearing and immunostaining. Sci. Rep. 15, 35101. 10.1038/s41598-025-18928-5 41062556 PMC12508232

[B11] Choquet D. Sainlos M. Sibarita J.-B. (2021). Advanced imaging and labelling methods to decipher brain cell organization and function. Nat. Rev. Neurosci. 22, 237–255. 10.1038/s41583-021-00441-z 33712727

[B12] Chudakov D. M. Matz M. V. Lukyanov S. Lukyanov K. A. (2010). Fluorescent proteins and their applications in imaging living cells and tissues. Physiol. Rev. 90, 1103–1163. 10.1152/physrev.00038.2009 20664080

[B13] Chung K. Wallace J. Kim S.-Y. Kalyanasundaram S. Andalman A. S. Davidson T. J. (2013). Structural and molecular interrogation of intact biological systems. Nature 497, 332–337. 10.1038/nature12107 23575631 PMC4092167

[B14] Chwastowicz A. Wolny A. Sobień M. Barański M. Tomczuk J. Szatkowski M. (2025). Use of bovine serum albumin might impair immunofluorescence signal in thick tissue samples. Sci. Rep. 15, 21920. 10.1038/s41598-025-06876-z 40594992 PMC12215187

[B15] Condado-Morales I. Dingfelder F. Waibel I. Turnbull O. M. Patel B. Cao Z. (2024). A comparative study of the developability of full-length antibodies, fragments, and bispecific formats reveals higher stability risks for engineered constructs. MAbs 16, 2403156. 10.1080/19420862.2024.2403156 39364796 PMC11457596

[B16] Crivianu-Gaita V. Thompson M. (2016). Aptamers, antibody scFv, and antibody fab' fragments: an overview and comparison of three of the Most versatile biosensor biorecognition elements. Biosens. Bioelectron. 85, 32–45. 10.1016/j.bios.2016.04.091 27155114

[B17] Croce A. C. Bottiroli G. (2017). Autofluorescence spectroscopy for monitoring metabolism in animal cells and tissues. Methods Mol. Biol. 1560, 15–43. 10.1007/978-1-4939-6788-9_2 28155143

[B18] Dean K. M. Palmer A. E. (2014). Advances in fluorescence labeling strategies for dynamic cellular imaging. Nat. Chem. Biol. 10, 512–523. 10.1038/nchembio.1556 24937069 PMC4248787

[B19] Dekkers J. F. Alieva M. Wellens L. M. Ariese H. C. R. Jamieson P. R. Vonk A. M. (2019). High-resolution 3D imaging of fixed and cleared organoids. Nat. Protoc. 14, 1756–1771. 10.1038/s41596-019-0160-8 31053799

[B20] Deliolanis N. C. Ale A. Morscher S. Burton N. C. Schaefer K. Radrich K. (2014). Deep-tissue reporter-gene imaging with fluorescence and optoacoustic tomography: a performance overview. Mol. Imaging Biol. 16, 652–660. 10.1007/s11307-014-0728-1 24609633

[B21] Deng Y. Xu J. Yu T. Zhu D. (2025). Advances in tissue optical clearing for 3D imaging in large animal. Front. Optoelectron. 18, 18. 10.1007/s12200-025-00162-6 40824479 PMC12361029

[B22] Deverman B. E. Pravdo P. L. Simpson B. P. Kumar S. R. Chan K. Y. Banerjee A. (2016). Cre-dependent selection yields AAV variants for widespread gene transfer to the adult brain. Nat. Biotechnol. 34, 204–209. 10.1038/nbt.3440 26829320 PMC5088052

[B23] Dodt H.-U. Leischner U. Schierloh A. Jährling N. Mauch C. P. Deininger K. (2007). Ultramicroscopy: three-dimensional visualization of neuronal networks in the whole mouse brain. Nat. Methods 4, 331–336. 10.1038/nmeth1036 17384643

[B24] Du H. Hou P. Wang L. Wang Z. Li Q. (2019). Modified CLARITY achieving faster and better intact mouse brain clearing and immunostaining. Sci. Rep. 9, 10571. 10.1038/s41598-019-46814-4 31332235 PMC6646319

[B25] Dwyer J. Ramirez M. D. Katz P. S. Karlstrom R. O. Bergan J. (2021). Accelerated clearing and molecular labeling of biological tissues using magnetohydrodynamic force. Sci. Rep. 11, 16462. 10.1038/s41598-021-95692-2 34385489 PMC8360944

[B26] Eisenberg J. D. Bradley R. P. Graham K. D. Ceron R. H. Lemke A. M. Wilkins B. J. (2024). Three-dimensional imaging of the enteric nervous system in human pediatric Colon reveals new features of hirschsprung’s disease. Gastroenterology 167, 547–559. 10.1053/j.gastro.2024.02.045 38494035 PMC11260536

[B27] Ertürk A. (2024). Deep 3D histology powered by tissue clearing, omics and AI. Nat. Methods 21, 1153–1165. 10.1038/s41592-024-02327-1 38997593

[B28] Ertürk A. Becker K. Jährling N. Mauch C. P. Hojer C. D. Egen J. G. (2012). Three-dimensional imaging of solvent-cleared organs using 3DISCO. Nat. Protoc. 7, 1983–1995. 10.1038/nprot.2012.119 23060243

[B29] Exbrayat J. M. (2019). Some aspects of the history of histology and evolution of the microscope in biomedical sciences. J. Histol. Histopathol. Res. 3 (1), 5–31.

[B30] Feng R. Xie J. Gao L. (2024). EDTP enhances and protects the fluorescent signal of GFP in cleared and expanded tissues. Sci. Rep. 14, 15279. 10.1038/s41598-024-66398-y 38961181 PMC11222453

[B31] Fumoto S. Kinoshita E. Ohta K. Nakamura K. Hirayama T. Nagasawa H. (2020). A pH-Adjustable tissue clearing solution that preserves lipid ultrastructures: suitable tissue clearing method for DDS evaluation. Pharmaceutics 12, 1070. 10.3390/pharmaceutics12111070 33182398 PMC7698078

[B32] Funk E. M. Jafree D. J. Hansmeier N. R. Abad Baucells C. Behncke R. Y. Pomeranz G. (2025). Nanobody immunolabelling and three-dimensional imaging reveals spatially restricted LYVE1 expression by kidney lymphatic vessels in mice. J. Nanobiotechnol 23, 667. 10.1186/s12951-025-03759-3 PMC12519744 41084009

[B33] Gradinaru V. Treweek J. Overton K. Deisseroth K. (2018). Hydrogel-tissue chemistry: principles and applications. Annu. Rev. Biophys. 47, 355–376. 10.1146/annurev-biophys-070317-032905 29792820 PMC6359929

[B34] Guo Z. Zheng Y. Zhang Y. (2022). CLARITY techniques based tissue clearing: types and differences. Folia Morphol. (Warsz.) 81, 1–12. 10.5603/FM.a2021.0012 33577077

[B35] Harrison A. S. Bjarkadottir B. D. Kuscu N. Davies J. Lane S. Williams S. A. (2025). Three-dimensional visualisation of human ovarian follicles using a whole-mount immunolabelling and optical tissue clearing method. Reprod. Fertil. 6, e250096. 10.1530/RAF-25-0096 41171604 PMC12957915

[B36] Hazart D. Delhomme B. Oheim M. Ricard C. (2023). Label-free, fast, 2-photon volume imaging of the organization of neurons and glia in the enteric nervous system. Front. Neuroanat. 16, 1070062. 10.3389/fnana.2022.1070062 36844894 PMC9948619

[B37] Hofmann J. Gadjalova I. Mishra R. Ruland J. Keppler S. J. (2021). Efficient tissue clearing and multi-organ volumetric imaging enable quantitative visualization of sparse immune cell populations during inflammation. Front. Immunol. 25, 599495. 10.3389/fimmu.2020.599495 33569052 PMC7869862

[B38] Horenberg A. L. Ren Y. Zeng E. Z. Rindone A. N. Pathak A. P. Grayson W. L. (2025). 3D imaging reveals changes in the neurovascular architecture of the murine calvarium with aging. Bone Res. 13, 24. 10.1038/s41413-025-00401-8 39984434 PMC11845787

[B39] Inagaki S. Nakagawa-Tamagawa N. Huynh N. Z. Kambe Y. Yagasaki R. Manita S. (2026). Isotonic and minimally invasive optical clearing media for live cell imaging *ex vivo* and *in vivo* . Nat. Methods 23, 839–853. 10.1038/s41592-026-03023-y 41820664 PMC13076227

[B40] Jin B.-H. Woo J. Lee M. Ku S. Moon H. S. Ryu S. J. (2023). Optimization of the optical transparency of bones by PACT-Based passive tissue clearing. Exp. Mol. Med. 55, 2190–2204. 10.1038/s12276-023-01089-8 37779150 PMC10618275

[B41] Jing D. Zhang S. Luo W. Gao X. Men Y. Ma C. (2018). Tissue clearing of both hard and soft tissue organs with the PEGASOS method. Cell. Res. 28, 803–818. 10.1038/s41422-018-0049-z 29844583 PMC6082844

[B42] Jing D. Yi Y. Luo W. Zhang S. Yuan Q. Wang J. (2019). Tissue clearing and its application to bone and dental tissues. J. Dent. Res. 98, 621–631. 10.1177/0022034519844510 31009584 PMC6535919

[B43] Ke M.-T. Fujimoto S. Imai T. (2013). SeeDB: a simple and morphology-preserving optical clearing agent for neuronal circuit reconstruction. Nat. Neurosci. 16, 1154–1161. 10.1038/nn.3447 23792946

[B44] Kiemen A. L. Damanakis A. I. Braxton A. M. He J. Laheru D. Fishman E. K. (2023). Tissue clearing and 3D reconstruction of digitized, serially sectioned slides provide novel insights into pancreatic cancer. Med 4, 75–91. 10.1016/j.medj.2022.11.009 36773599 PMC9922376

[B45] Kim S.-Y. Cho J. H. Murray E. Bakh N. Choi H. Ohn K. (2015). Stochastic electrotransport selectively enhances the transport of highly electromobile molecules. Proc. Natl. Acad. Sci. U.S.A. 112, E6274–E6283. 10.1073/pnas.1510133112 26578787 PMC4655572

[B46] Kohl J. Ng J. Cachero S. Ciabatti E. Dolan M.-J. Sutcliffe B. (2014). Ultrafast tissue staining with chemical tags. Proc. Natl. Acad. Sci. U.S.A. 111, E3805–E3814. 10.1073/pnas.1411087111 25157152 PMC4246963

[B47] Kosmidis S. Negrean A. Dranovsky A. Losonczy A. Kandel E. R. (2021). A fast, aqueous, reversible three-day tissue clearing method for adult and embryonic mouse brain and whole body. Cell. Rep. Methods 1, 100090. 10.1016/j.crmeth.2021.100090 34966901 PMC8713566

[B48] Ku T. Guan W. Evans N. B. Sohn C. H. Albanese A. Kim J.-G. (2020). Elasticizing tissues for reversible shape transformation and accelerated molecular labeling. Nat. Methods 17, 609–613. 10.1038/s41592-020-0823-y 32424271 PMC8056749

[B49] Kumar V. Krolewski D. M. Hebda-Bauer E. K. Parsegian A. Martin B. Foltz M. (2021). Optimization and evaluation of fluorescence *in situ* hybridization chain reaction in cleared fresh-frozen brain tissues. Brain Struct. Funct. 226, 481–499. 10.1007/s00429-020-02194-4 33386994 PMC7962668

[B50] Lai H. M. Ng W. L. Gentleman S. M. Wu W. (2017). Chemical probes for visualizing intact animal and human brain tissue. Cell. Chem. Biol. 22, 659–672. 10.1016/j.chembiol.2017.05.015 28644957

[B51] Lai H. M. Tang Y. Lau Z. Y. H. Campbell R. A. A. Yau J. C. N. Chan C. C. Y. (2022). Antibody stabilization for thermally accelerated deep immunostaining. Nat. Methods 19, 1137–1146. 10.1038/s41592-022-01569-1 36050489 PMC9467915

[B52] Lallemant L. Lebreton C. Garfa-Traoré M. (2020). Comparison of different clearing and acquisition methods for 3D imaging of murine intestinal organoids. J. Biol. Methods 7, e141. 10.14440/jbm.2020.334 33564693 PMC7865078

[B53] Laurino A. Franceschini A. Pesce L. Cinci L. Montalbano A. Mazzamuto G. (2023). A guide to perform 3D histology of biological tissues with fluorescence microscopy. Int. J. Mol. Sci. 24, 6747. 10.3390/ijms24076747 37047724 PMC10094801

[B54] Laxman P. Ansari S. Gaus K. Goyette J. (2021). The benefits of unnatural amino acid incorporation as protein labels for single molecule localization microscopy. Front. Chem. 9, 641355. 10.3389/fchem.2021.641355 33842432 PMC8027105

[B55] Lazutkin A. A. Shuvaev S. A. Barykina N. V. (2019). Click histochemistry for whole-mount staining of brain structures. MethodsX 6, 1986–1991. 10.1016/j.mex.2019.09.011 31667095 PMC6812327

[B56] Lee E. Choi J. Jo Y. Kim J. Y. Jang Y. J. Lee H. M. (2016). ACT-PRESTO: rapid and consistent tissue clearing and labeling method for 3-dimensional (3D) imaging. Sci. Rep. 6, 18631. 10.1038/srep18631 26750588 PMC4707495

[B57] Lee B. Lee E. Kim J. H. Kim H.-J. Kang Y. G. Kim H. J. (2021). Sensitive label-free imaging of brain samples using FxClear-based tissue clearing technique. iScience 24, 102267. 10.1016/j.isci.2021.102267 33817573 PMC8005756

[B59] Li Y. Xu J. Wan P. Yu T. Zhu D. (2018). Optimization of GFP fluorescence preservation by a modified uDISCO clearing protocol. Front. Neuroanat. 12, 67. 10.3389/fnana.2018.00067 30158858 PMC6104128

[B60] Li W. Germain R. N. Gerner M. Y. (2019). High-dimensional cell-level analysis of tissues with Ce3D multiplex volume imaging. Nat. Protoc. 14, 1708–1733. 10.1038/s41596-019-0156-4 31028373 PMC8690297

[B61] Li X. Kot J. C. K. Tsang V. T. C. Lo C. T. K. Huang B. Tian Y. (2022). Ultraviolet photoacoustic microscopy with tissue clearing for high-contrast histological imaging. Photoacoustics 25, 100313. 10.1016/j.pacs.2021.100313 34804794 PMC8581572

[B62] Li J. He L. Wang W. Wang S. Zhang D. Liang L. (2025). Comprehensive evaluation and application of tissue clearing techniques for 3-D visualization of splenic neural and immune architecture. Am. J. Physiology-Cell Physiology 328, C1699–C1715. 10.1152/ajpcell.00084.2025 40249862

[B63] Lin Y.-H. Wang L.-W. Chen Y.-H. Chan Y.-C. Hu S.-H. Wu S.-Y. (2024). Revealing intact neuronal circuitry in centimeter-sized formalin-fixed paraffin-embedded brain. eLife 13, RP93212. 10.7554/eLife.93212 38775133 PMC11111220

[B64] Liu L. Chen A. Li Y. Mulder J. Heyn H. Xu X. (2024). Spatiotemporal omics for biology and medicine. Cell. 187, 4488–4519. 10.1016/j.cell.2024.07.040 39178830

[B65] Lopes M. M. Paysan J. Rino J. Lopes S. M. Pereira De Almeida L. Cortes L. (2022). A new protocol for whole-brain biodistribution analysis of AAVs by tissue clearing, light-sheet microscopy and semi-automated spatial quantification. Gene Ther. 29, 665–679. 10.1038/s41434-022-00372-z 36316447

[B66] Lotze J. Reinhardt U. Seitz O. Beck-Sickinger A. G. (2016). Peptide-tags for site-specific protein labelling *in vitro* and *in vivo* . Mol. Biosyst. 12, 1731–1745. 10.1039/C6MB00023A 26960991

[B67] Luo J. Molbay M. Chen Y. Horvath I. Kadletz K. Kick B. (2025). Nanocarrier imaging at single-cell resolution across entire mouse bodies with deep learning. Nat. Biotechnol. 43, 2009–2022. 10.1038/s41587-024-02528-1 39809933 PMC12700832

[B68] Mai H. Rong Z. Zhao S. Cai R. Steinke H. Bechmann I. (2022). Scalable tissue labeling and clearing of intact human organs. Nat. Protoc. 17, 2188–2215. 10.1038/s41596-022-00712-8 35859136

[B69] Mai H. Luo J. Hoeher L. Al-Maskari R. Horvath I. Chen Y. (2024). Whole-body cellular mapping in mouse using standard IgG antibodies. Nat. Biotechnol. 42, 617–627. 10.1038/s41587-023-01846-0 37430076 PMC11021200

[B70] Makki M. Molander Z. A. Pineda-Castillo S. A. Laurence D. W. Singhal S. Eltwafsha Y. (2025). Evaluation of the effects of clearing agents, fixation, and process durations on cardiovascular tissue imaging with second harmonic generation and multi-photon modalities. Front. Bioeng. Biotechnol. 13, 1606425. 10.3389/fbioe.2025.1606425 40787195 PMC12331724

[B145] Mao C. Lee M. Y. Jhan J. R. Halpern A. R. Woodworth M. A. Glaser A. K. (2020). Feature-rich covalent stains for super-resolution and cleared tissue fluorescence microscopy. Sci. Adv. 6, eaba4542. 10.1126/sciadv.aba4542 32518827 PMC7253160

[B71] Matsumoto K. Mitani T. T. Horiguchi S. A. Kaneshiro J. Murakami T. C. Mano T. (2019). Advanced CUBIC tissue clearing for whole-organ cell profiling. Nat. Protoc. 14, 3506–3537. 10.1038/s41596-019-0240-9 31748753

[B72] Mayorca-Guiliani A. E. Willacy O. Madsen C. D. Rafaeva M. Elisabeth Heumüller S. Bock F. (2019). Decellularization and antibody staining of mouse tissues to map native extracellular matrix structures in 3D. Nat. Protoc. 14, 3395–3425. 10.1038/s41596-019-0225-8 31705125

[B73] Meng L. Song X. Wang J. Shi W. Gao L. Jiang X. (2025). Tissue clearing and its application in dental research. BMEMat 3, e12113. 10.1002/bmm2.12113

[B74] Messal H. A. Almagro J. Zaw Thin M. Tedeschi A. Ciccarelli A. Blackie L. (2021). Antigen retrieval and clearing for whole-organ immunofluorescence by FLASH. Nat. Protoc. 16, 239–262. 10.1038/s41596-020-00414-z 33247285

[B75] Mino T. Nonaka H. Hamachi I. (2024). Molecular anchoring and fluorescent labeling in animals compatible with tissue clearing for 3D imaging. Curr. Opin. Chem. Biol. 81, 102474. 10.1016/j.cbpa.2024.102474 38838505

[B76] Molbay M. Kolabas Z. I. Todorov M. I. Ohn T. L. Ertürk A. (2021). A guidebook for DISCO tissue clearing. Mol. Syst. Biol. 17, e9807. 10.15252/msb.20209807 33769689 PMC7995442

[B77] Murray E. Cho J. H. Goodwin D. Ku T. Swaney J. Kim S. Y. (2015). Simple, scalable proteomic imaging for high-dimensional profiling of intact systems. Cell. 163, 1500–1514. 10.1016/j.cell.2015.11.025 26638076 PMC5275966

[B78] Na M. Kim K. Lim H. R. Ha C. M. Chang S. (2021). Rapid immunostaining method for three-dimensional volume imaging of biological tissues by magnetic force-induced focusing of the electric field. Brain Struct. Funct. 226, 297–309. 10.1007/s00429-020-02160-0 33175320

[B79] Na M. Kim K. Oh K. Choi H. J. Ha C. Chang S. (2022). Sodium cholate-based active delipidation for rapid and efficient clearing and immunostaining of deep biological samples. Small Methods 6, e2100943. 10.1002/smtd.202100943 35041279

[B80] Ngo T. B. DeStefano S. Liu J. Su Y. Shroff H. Vishwasrao H. D. (2023). Label‐free cleared tissue microscopy and machine learning for 3D histopathology of biomaterial implants. J. Biomed. Mater. Res. 111, 840–850. 10.1002/jbm.a.37515 PMC12316057 36861434

[B81] Nguyen L. Dohi T. Watanabe‐Takano H. Fukuhara S. Ogawa R. (2025). Comprehensive analysis of keloid vasculature by tissue clearing and 3D imaging. Wound Repair Regen. 33, e70015. 10.1111/wrr.70015 40143403 PMC11947297

[B82] Novoselova M. V. Abakumova T. O. Khlebtsov B. N. Zatsepin T. S. Lazareva E. N. Tuchin V. V. (2020). Optical clearing for photoacoustic lympho- and angiography beyond conventional depth limit *in vivo* . Photoacoustics 20, 100186. 10.1016/j.pacs.2020.100186 32637316 PMC7327268

[B83] Özen I. Mai H. De Maio A. Ruscher K. Michalettos G. Clausen F. (2022). Purkinje cell vulnerability induced by diffuse traumatic brain injury is linked to disruption of long-range neuronal circuits. Acta Neuropathol. Commun. 10, 129. 10.1186/s40478-022-01435-3 36064443 PMC9446851

[B84] Paavolainen O. Peurla M. Koskinen L. M. Pohjankukka J. Saberi K. Tammelin E. (2024). Volumetric analysis of the terminal ductal lobular unit architecture and cell phenotypes in the human breast. Cell. Rep. 43, 114837. 10.1016/j.celrep.2024.114837 39368089

[B85] Pan C. Schoppe O. Parra-Damas A. Cai R. Todorov M. I. Gondi G. (2019). Deep learning reveals cancer metastasis and therapeutic antibody targeting in the entire body. Cell. 179, 1661–1676.e19. 10.1016/j.cell.2019.11.013 31835038 PMC7591821

[B86] Pang Z. Schafroth M. A. Ogasawara D. Wang Y. Nudell V. Lal N. K. (2022). *In situ* identification of cellular drug targets in Mammalian tissue. Cell. 185, 1793–1805.e17. 10.1016/j.cell.2022.03.040 35483372 PMC9106931

[B87] Park Y. G. Sohn C. H. Chen R. McCue M. Yun D. H. Drummond G. T. (2019). Protection of tissue physicochemical properties using polyfunctional crosslinkers. Nat. Biotechnol. 37, 73–83. 10.1038/nbt.4281 30556815 PMC6579717

[B88] Pesce L. Scardigli M. Gavryusev V. Laurino A. Mazzamuto G. Brady N. (2022). 3D molecular phenotyping of cleared human brain tissues with light-sheet fluorescence microscopy. Commun. Biol. 5, 447. 10.1038/s42003-022-03390-0 35551498 PMC9098858

[B89] Pichon J. Ledevin M. Larcher T. Jamme F. Rouger K. Dubreil L. (2022). Label‐free 3D characterization of cardiac fibrosis in muscular dystrophy using SHG imaging of cleared tissue. Biol. Cell. 114, 91–103. 10.1111/boc.202100056 34964145

[B90] Porzberg N. Gries K. Johnsson K. (2025). Exploiting covalent chemical labeling with self-labeling proteins. Annu. Rev. Biochem. 94, 29–58. 10.1146/annurev-biochem-030222-121016 40106680

[B91] Puelles V. G. Combes A. N. Bertram J. F. (2021). Clearly imaging and quantifying the kidney in 3D. Kidney Int. 100, 780–786. 10.1016/j.kint.2021.04.042 34089762

[B92] Pulous F. E. Cruz-Hernández J. C. Yang C. Kaya Ζ. Paccalet A. Wojtkiewicz G. (2022). Cerebrospinal fluid can exit into the skull bone marrow and instruct cranial hematopoiesis in mice with bacterial meningitis. Nat. Neurosci. 25, 567–576. 10.1038/s41593-022-01060-2 35501382 PMC9081225

[B93] Qi Y. Yu T. Xu J. Wan P. Ma Y. Zhu J. (2019). FDISCO: advanced solvent-based clearing method for imaging whole organs. Sci. Adv. 5, eaau8355. 10.1126/sciadv.aau8355 30746463 PMC6357753

[B94] Ren J. Choi H. Chung K. Bouma B. E. (2017). Label-free volumetric optical imaging of intact murine brains. Sci. Rep. 7, 46306. 10.1038/srep46306 28401897 PMC5388920

[B95] Renier N. Wu Z. Simon D. J. Yang J. Ariel P. Tessier-Lavigne M. (2014). iDISCO: a simple, rapid method to immunolabel large tissue samples for volume imaging. Cell. 159, 896–910. 10.1016/j.cell.2014.10.010 25417164

[B96] Richardson D. S. Lichtman J. W. (2015). Clarifying tissue clearing. Cell. 162, 246–257. 10.1016/j.cell.2015.06.067 26186186 PMC4537058

[B97] Richardson D. S. Guan W. Matsumoto K. Pan C. Chung K. Ertürk A. (2021). Tissue clearing. Nat. Rev. Methods Prim. 1, 84. 10.1038/s43586-021-00080-9 PMC8815095 35128463

[B98] Rong Z. Ertürk A. Tang Y. Mai H. (2025). Tissue clearing and its application in nanoparticle development. Small 21, e2410032. 10.1002/smll.202410032 39901464

[B99] Rosen G. A. Kirsch D. Nicks R. Kelley H. Mathias R. Cormier K. A. (2024). SHARD: an improved method for staining and visualizing multiplex immunofluorescence in optically cleared postmortem human brain tissue. Front. Neurosci. 18, 1474617. 10.3389/fnins.2024.1474617 39445075 PMC11496292

[B100] Sakaguchi R. Leiwe M. N. Imai T. (2018). Bright multicolor labeling of neuronal circuits with fluorescent proteins and chemical tags. eLife 7, e40350. 10.7554/eLife.40350 30454553 PMC6245733

[B101] Sakamoto D. M. Tamura I. Yi B. Hasegawa S. Saito Y. Yamada N. (2024). Whole-body and whole-organ 3D imaging of hypoxia using an activatable covalent fluorescent probe compatible with tissue clearing. ACS Nano 18, 5167–5179. 10.1021/acsnano.3c12716 38301048

[B102] Schneidereit D. Bröllochs A. Ritter P. Kreiß L. Mokhtari Z. Beilhack A. (2021). An advanced optical clearing protocol allows label-free detection of tissue necrosis *via* multiphoton microscopy in injured whole muscle. Theranostics 11, 2876–2891. 10.7150/thno.51558 33456578 PMC7806485

[B103] Seike N. Kojima R. Tachibana R. Fujita K. Yanagisawa T. Yokoyama S. (2025). Genetic code expansion-driven *in situ* click labeling enables rapid imaging-based selection of functional nanobody–dye conjugates. Chem. Pharm. Bull. 73, 793–801. 10.1248/cpb.c25-00376 40915908

[B104] Seo J. Choe M. Kim S.-Y. (2016). Clearing and labeling techniques for large-scale biological tissues. Mol. Cells 39, 439–446. 10.14348/molcells.2016.0088 27239813 PMC4916395

[B105] Shan Q. H. Qin X. Y. Zhou N. Huang C. Wang Y. Chen P. (2022). A method for ultrafast tissue clearing that preserves fluorescence for multimodal and longitudinal brain imaging. BMC Biol. 29, 77. 10.1186/s12915-022-01275-6 PMC8966190 35351101

[B106] Shi L. Wei M. Miao Y. Qian N. Shi L. Singer R. A. (2022). Highly-multiplexed volumetric mapping with raman dye imaging and tissue clearing. Nat. Biotechnol. 40, 364–373. 10.1038/s41587-021-01041-z 34608326 PMC8930416

[B107] Smith S. N. Schubert R. Simic B. Brücher D. Schmid M. Kirk N. (2021). The SHREAD gene therapy platform for paracrine delivery improves tumor localization and intratumoral effects of a clinical antibody. Proc. Natl. Acad. Sci. U.S.A. 118, e2017925118. 10.1073/pnas.2017925118 34001602 PMC8166199

[B108] Stoecker A. Pinkert-Leetsch D. Koch T. Ackermann R. Nolte S. Van Oterendorp C. (2024). Collagen crosslinking-induced corneal morphological changes: a three-dimensional light sheet Microscopy-based evaluation. Sci. Rep. 14, 28330. 10.1038/s41598-024-78516-x 39550403 PMC11569206

[B109] Streit M. Budiarta M. Jungblut M. Beliu G. (2025). Fluorescent labeling strategies for molecular bioimaging. Biophys. Rep. 5, 100200. 10.1016/j.bpr.2025.100200 PMC11914189 39947326

[B110] Sun H. Meng S. Xu Z. Cai H. Pei X. Wan Q. (2023). Vascular and lymphatic heterogeneity and age-related variations of dental pulps. J. Dent. 138, 104695. 10.1016/j.jdent.2023.104695 37714450

[B111] Susaki E. A. Tainaka K. Perrin D. Kishino F. Tawara T. Watanabe T. M. (2014). Whole-brain imaging with single-cell resolution using chemical cocktails and computational analysis. Cell. 157, 726–739. 10.1016/j.cell.2014.03.042 24746791

[B112] Tainaka K. Kuno A. Kubota S. I. Murakami T. Ueda H. R. (2016). Chemical principles in tissue clearing and staining protocols for whole-body cell profiling. Annu. Rev. Cell. Dev. Biol. 32, 713–741. 10.1146/annurev-cellbio-111315-125001 27298088

[B113] Tamura I. Sakamoto D. M. Yi B. Saito Y. Yamada N. Morimoto J. (2024). Click3D: click reaction across deep tissues for whole-organ 3D fluorescence imaging. Sci. Adv. 10, eado8471. 10.1126/sciadv.ado8471 39018410 PMC466963

[B114] Thai J. Fuller‐Jackson J. Ivanusic J. J. (2024). Using tissue clearing and light sheet fluorescence microscopy for the three‐dimensional analysis of sensory and sympathetic nerve endings that innervate bone and dental tissue of mice. J Comp. Neurology 532, e25582. 10.1002/cne.25582 PMC10952626 38289188

[B115] Tian T. Yang Z. Li X. (2021). Tissue clearing technique: recent progress and biomedical applications. J. Anat. 238, 489–507. 10.1111/joa.13309 32939792 PMC7812135

[B116] Touloumes G. J. Ardoña H. A. M. Casalino E. K. Zimmerman J. F. Chantre C. O. Bitounis D. (2021). Mapping 2D- and 3D-distributions of metal/metal Oxide Nanoparticles Within Cleared Human Ex Vivo Skin Tissues. 10.1016/j.impact.2020.100208 PMC7687853 33251378

[B117] Treweek J. B. Chan K. Y. Flytzanis N. C. Yang B. Deverman B. E. Greenbaum A. (2015). Whole-body tissue stabilization and selective extractions via tissue-hydrogel hybrids for high-resolution intact circuit mapping and phenotyping. Nat. Protoc. 10, 1860–1896. 10.1038/nprot.2015.122 26492141 PMC4917295

[B118] Uchinomiya S. Ojida A. Hamachi I. (2014). Peptide tag/probe pairs based on the coordination chemistry for protein labeling. Inorg. Chem. 53, 1816–1823. 10.1021/ic401612z 24131471

[B119] Ueda H. R. Ertürk A. Chung K. Gradinaru V. Chédotal A. Tomancak P. (2020). Tissue clearing and its applications in neuroscience. Nat. Rev. Neurosci. 21, 61–79. 10.1038/s41583-019-0250-1 31896771 PMC8121164

[B120] Wan P. Li Y. Zhu J. Xu J. Liu X. Yu T. (2021). FDISCO+: a clearing method for robust fluorescence preservation of cleared samples. Neurophotonics 8, 035007. 10.1117/1.NPh.8.3.035007 34514032 PMC8427119

[B121] Wang X. Allen W. E. Wright M. A. Sylwestrak E. L. Samusik N. Vesuna S. (2018). Three-dimensional intact-tissue sequencing of single-cell transcriptional states. Science 361, eaat5691. 10.1126/science.aat5691 29930089 PMC6339868

[B122] Wang Y. Eddison M. Fleishman G. Weigert M. Xu S. Wang T. (2021b). EASI-FISH for thick tissue defines lateral hypothalamus spatio-molecular organization. Cell. 184, 6361–6377.e24. 10.1016/j.cell.2021.11.024 34875226

[B123] Wang W. Zhang N. Du Y. Gao J. Li M. Lin L. (2021a). Three‐dimensional quantitative imaging of native microbiota distribution in the gut. Angew. Chem. Int. Ed. 60, 3055–3061. 10.1002/anie.202010921 33084179

[B124] Wang K. Yu Y. Xu Y. Yue Y. Zhao F. Feng W. (2023). TSA-PACT: a method for tissue clearing and immunofluorescence staining on zebrafish brain with improved sensitivity, specificity and stability. Cell. Biosci. 13, 97. 10.1186/s13578-023-01043-1 37237300 PMC10223841

[B125] Wang Y. Jiang Z. Zhang K. Tang H. Wang G. Gao J. (2024). Whole‐tumor clearing and imaging of intratumor microbiota in three dimensions with miCDaL strategy. Adv. Sci. 11, 2400694. 10.1002/advs.202400694 PMC11600245 39378003

[B126] Wei M. Shi L. Shen Y. Zhao Z. Guzman A. Kaufman L. J. (2019). Volumetric chemical imaging by clearing-enhanced stimulated raman scattering microscopy. Proc. Natl. Acad. Sci. U.S.A. 116, 6608–6617. 10.1073/pnas.1813044116 30872474 PMC6452712

[B127] Wilhelm J. Kühn S. Tarnawski M. Gotthard G. Tünnermann J. Tänzer T. (2021). Kinetic and structural characterization of the self-labeling protein tags HaloTag7, SNAP-tag, and CLIP-Tag. Biochemistry 60, 2560–2575. 10.1021/acs.biochem.1c00258 34339177 PMC8388125

[B128] Woelfle S. Deshpande D. Feldengut S. Braak H. Del Tredici K. Roselli F. (2023). CLARITY increases sensitivity and specificity of fluorescence immunostaining in long-term archived human brain tissue. BMC Biol. 21, 113. 10.1186/s12915-023-01582-6 37221592 PMC10207789

[B129] Yamauchi K. Koike M. Hioki H. (2024). A Three Dimensional Immunolabeling Method with peroxidase-fused Nanobodies and Fluorochromized Tyramide-Glucose Oxidase Signal Amplification. 10.1101/2024.10.25.620157 PMC12177075 40533487

[B130] Yang B. Treweek J. B. Kulkarni R. P. Deverman B. E. Chen C. K. Lubeck E. (2014). Single-cell phenotyping within transparent intact tissue through whole-body clearing. Cell. 158, 945–958. 10.1016/j.cell.2014.07.017 25088144 PMC4153367

[B131] Yi Y. Men Y. Jing D. Luo W. Zhang S. Feng J. Q. (2019). 3‐dimensional visualization of implant‐tissue interface with the polyethylene glycol associated solvent system tissue clearing method. Cell. Prolif. 52, e12578. 10.1111/cpr.12578 30714253 PMC6536405

[B132] Yi Y. Li Y. Zhang S. Men Y. Wang Y. Jing D. (2024). Mapping of individual sensory nerve axons from digits to spinal cord with the transparent embedding solvent system. Cell. Res. 34, 124–139. 10.1038/s41422-023-00867-3 38168640 PMC10837210

[B133] Yi B. Yatabe H. Sakamoto D. M. Tamura I. Saito Y. Yamada N. (2025). Imaging heterogeneous patterns of aminopeptidase N activity in hierarchical tissue structures through high‐resolution whole‐organ 3D mapping. Angew. Chem. Int. Ed. 64, e202504668. 10.1002/anie.202504668 PMC12105696 40129052

[B134] Yoshida S. Y. Matsumoto K. Takagi S. Kinoshita F. L. Yamashita K. Shigeta D. (2026). Whole-organ and whole-body 3D atlases enable cellome-wide profiling. Cell. 189, 1836–1853.e19. 10.1016/j.cell.2025.12.057 41747726

[B135] Yu T. Qi Y. Zhu J. Xu J. Gong H. Luo Q. (2017). Elevated-temperature-induced acceleration of PACT clearing process of mouse brain tissue. Sci. Rep. 7, 38848. 10.1038/srep38848 28139694 PMC5282525

[B136] Yu T. Zhu J. Li D. Zhu D. (2021). Physical and chemical mechanisms of tissue optical clearing. iScience 43, 2031–2042. 10.1038/s41587-024-02533-4 PMC7920833 33718830

[B137] Yun D. H. Park Y. G. Cho J. H. Kamentsky L. Evans N. B. Albanese A. (2025). Uniform volumetric single-cell processing for organ-scale molecular phenotyping. Nat. Biotechnol. 43, 2031–2042. 10.1038/s41587-024-02533-4 39856430

[B138] Zhan Y. Wu H. Liu L. Lin J. Zhang S. (2021). Organic solvent‐based tissue clearing techniques and their applications. J. Biophot. 14, e202000413. 10.1002/jbio.202000413 33715302

[B139] Zhang M. Eichhorn S. W. Zingg B. Yao Z. Cotter K. Zeng H. (2021a). Spatially resolved cell atlas of the mouse primary motor cortex by MERFISH. Nature 598, 137–143. 10.1038/s41586-021-03705-x 34616063 PMC8494645

[B140] Zhang N. Li X. Czajkowsky D. M. Zhang H. Alam M. S. Shao Z. (2021b). Efficient and fast immuno-labeling of clarified tissues using low-field enhanced diffusion. IEEE Trans. Biomed. Eng. 68, 3301–3307. 10.1109/TBME.2021.3070146 33788676

[B141] Zhao S. Todorov M. I. Cai R. Maskari R. A. Steinke H. Kemter E. (2020). Cellular and molecular probing of intact human organs. Cell. 180, 796–812.e19. 10.1016/j.cell.2020.01.030 32059778 PMC7557154

[B142] Zhong S. Zhang X. Gao X. Li Z. Huang L. Guo Q. (2025). Ultrabright chemical labeling enables rapid neural connectivity profiling in large tissue samples. Neuron 113, 3741–3757.e11. 10.1016/j.neuron.2025.08.022 40972576

[B143] Zhu X. Huang L. Zheng Y. Song Y. Xu Q. Wang J. (2019). Ultrafast optical clearing method for three-dimensional imaging with cellular resolution. Proc. Natl. Acad. Sci. U.S.A. 116, 11480–11489. 10.1073/pnas.1819583116 31101714 PMC6561250

[B144] Zhu J. Liu X. Liu Z. Deng Y. Xu J. Liu K. (2024). SOLID: minimizing tissue distortion for brain-wide profiling of diverse architectures. Nat. Commun. 15, 8303. 10.1038/s41467-024-52560-7 39333107 PMC11436996

